# Sex Differences in Intimate Partner Violence Lethality Risk Screening Administration and Outcomes

**DOI:** 10.1001/jamanetworkopen.2025.45519

**Published:** 2025-12-03

**Authors:** Galina A. Portnoy, Elizabeth C. Coppola, Katherine M. Iverson, Melissa E. Dichter, Jacquelyn C. Campbell, LeAnn E. Bruce, Cynthia A. Brandt, Mark R. Relyea

**Affiliations:** 1 VA Connecticut Healthcare System, West Haven, Connecticut; 2 Yale School of Medicine, New Haven, Connecticut; 3 Women’s Health Sciences Division of the National Center for PTSD, VA Boston Healthcare System, Boston, Massachusetts; 4 Department of Psychiatry, Boston University Chobanian and Avedisian School of Medicine, Boston, Massachusetts; 5 VA Center for Health Equity Research and Promotion, Corporal Michael J. Crescenz VA Medical Center, Philadelphia, Pennsylvania; 6 School of Social Work, Temple University, Philadelphia, Pennsylvania; 7 Johns Hopkins University School of Nursing, Baltimore, Maryland; 8 Veterans Healthcare Administration Intimate Partner Violence Assistance Program, Washington, District of Columbia; 9 Western Kentucky University School of Social Work, Bowling Green

## Abstract

**Importance:**

Intimate partner violence (IPV) is a critical public health issue. Health care systems, including the Veterans Health Administration, routinely screen patients for IPV and conduct secondary screening for IPV-related lethality when IPV is detected. Despite studies investigating IPV screening and response in the health care system, there has been limited empirical work on the implementation of screening for IPV-related lethality, especially among men.

**Objective:**

To examine sex differences in IPV-related lethality screening administration and clinical outcomes.

**Design, Setting, and Participants:**

This retrospective cross-sectional study used electronic health record data from veterans screened for IPV at 135 Veterans Affairs medical centers between October 1, 2022, and September 30, 2023.

**Exposure:**

Intimate partner violence screening.

**Main Outcomes and Measures:**

The receipt of protocol-concordant IPV-related lethality screening was assessed and subsequent clinical outcomes examined. Mixed-effects logistic regression was used to evaluate associations between sex and outcome variables.

**Results:**

Among the 67 379 patients with IPV-positive screens who should have received the secondary IPV-related lethality screening per protocol, 55 482 (82.3%; mean [SD] age, 52.3 [16.1] years; 77.0% men) were administered the secondary screen, with the error rate in screening administration higher among men than women (adjusted odds ratio, 1.42 [95% CI, 1.33-1.51]). Of patients screened, 16.9% reported past-year IPV-related lethality risk. Women were significantly more likely to be at risk of IPV-related lethality compared with men (adjusted odds ratio, 2.29 [95% CI, 2.16-2.41]), with 29.9% of women and 13.3% of men reporting lethality risk.

**Conclusions and Relevance:**

This cross-sectional study of sex differences in IPV-related lethality screening found sex-based differences in the administration and clinical outcomes of IPV-related lethality screening among patients screened for IPV during health care encounters. Nearly 1 in 5 men were misclassified as negative for IPV and, therefore, not administered the IPV-related lethality screen per protocol. Among patients who received the second screen, lethality risk was significantly higher for women across all screening items compared with men. Further research is needed to assess IPV experiences among men, including gaps in health care and opportunities to improve implementation of IPV screening and response for men, as well as to identify effective strategies for connecting all patients at risk of lethal IPV to lifesaving services.

## Key Points

**Question:**

What are the patterns of intimate partner violence (IPV) lethality risk screening administration and outcomes among Veterans Health Administration patients?

**Findings:**

In this cross-sectional study of 55 482 patients screened positive for IPV across 135 Veterans Affairs medical centers, 9354 (16.9%) screened positive for IPV-related lethality risk, with women at higher risk than men. However, men were significantly more likely to be misclassified as IPV negative during screening and not administered the subsequent IPV-related lethality screen.

**Meaning:**

These findings suggest that many patients at high risk for IPV-related lethality, especially men, may be missed in screening due to protocol errors and subsequently not connected to critical services and support.

## Teaser

This cross-sectional study examines sex differences in intimate partner violence (IPV)–related lethality screening administration and clinical outcomes among US veterans.

## Introduction

Intimate partner violence (IPV), defined as psychological, physical, or sexual harm by a current or former partner or spouse,^1^ is an important public health issue with far-reaching adverse outcomes. Health care settings serve as critical access points for IPV detection and follow-up care,^1^ particularly as IPV is associated with physical, psychological, and chronic health conditions. Moreover, patients with a history of IPV use the health care system at higher rates than those without IPV experiences.^2,3,4,5,6,7^ Accordingly, leading health organizations, such as the US Preventive Services Task Force (USPSTF), recommend universal screening and response for IPV among women of reproductive age in the health care system.^8,9^

The Veterans Health Administration (VHA) is the largest integrated health care system in the US, serving more than 6 million patients annually (91% male).^10^ Lessons learned from implementation of screening for IPV experiences in the VHA could guide screening implementation efforts across other health care settings. The VHA adopted the USPSTF’s IPV screening recommendations,^8^ which require annual screening of past-year IPV among women of reproductive age using a modified Hurt, Insult, Threaten, Scream (HITS) screening instrument^11,12,13^ and responding to positive screening results with a secondary screening for risk of potentially lethal IPV,^14,15^ education, and referral to care, as needed.^15,16,17^

Research on the implementation of IPV screening and response in VHA has primarily focused on women,^16,18,19,20,21^ reflecting historical priorities in the field and guidelines set by the USPSTF. However, emerging evidence has indicated that male veterans experience past-year IPV at rates comparable to their female counterparts (28.3% and 27.9%, respectively).^22^ Little is known about the implementation of IPV screening and response among men, including potential sex differences in administration of the screening protocol and clinicians’ responses to IPV disclosures.

Understanding sex differences in the implementation of IPV screening is crucial for developing and integrating equitable screening protocols, tailoring education and training, and clarifying expectations for screening across the health care system. Men who experience IPV often face barriers to reporting and receiving recognition and support within health care settings and may minimize their abuse or express concerns about not being taken seriously.^23,24^ Additionally, men encounter barriers to help-seeking, including stigma, ridicule, and blame from those to whom they disclose their experiences.^25,26,27^ Consequently, IPV among men is often underreported, unrecognized, and inadequately addressed in clinical settings.^28^ Research is needed to systematically investigate IPV screening procedures in health care settings, such as fidelity to IPV screening guidelines and protocols, including among men. Ensuring that lethality screenings are performed with fidelity to guidelines is especially important, as these tools identify individuals at the highest risk of severe violence and IPV-related lethality.^14,29^ Accordingly, our study aimed to investigate potential sex differences in (1) IPV-related lethality screening administration among patients who reported past-year IPV experiences and (2) lethality screening clinical outcomes and responses.

## Methods

### Data Sources and Study Population

This cross-sectional study drew data from the PRISM (Partnered Evaluation of Relationship Health Innovations and Services Through Mixed Methods) cohort,^30^ a national, retrospective cohort of approximately 6 million US veterans eligible for IPV screening based on their enrollment in VHA and at least 1 outpatient health care encounter during the observation period. Data from all VHA patients who completed IPV screening between October 1, 2022, and September 30, 2023 (N = 1 332 532) at 135 US Department of Veterans Affairs (VA) medical centers were extracted from the VA’s Corporate Data Warehouse, a centralized repository of electronic health record (EHR) data across VA medical centers and satellite clinics.^31^

Research using data collected as part of this quality improvement initiative was approved by the VA Connecticut Healthcare System Institutional Review Board. As our project only used deidentified EHR data, the Institutional Review Board waived the requirement for participant informed consent. We followed the Strengthening the Reporting of Observational Studies in Epidemiology (STROBE) reporting guideline for cross-sectional studies.^32^

### Main Outcomes and Measures

Past-year IPV was determined using the modified HITS,^11,12,13^ a 5-item screening tool that asks patients to indicate the frequency of which a partner engaged in any of the following in the past year: “scream or curse at you”; “insult or talk down to you”; “threaten you with harm”; “physically hurt you”; or “force or pressure you to have sexual contact against your will, or when you were unable to say no.” Per VHA policy, a positive endorsement of any item on this primary screen is considered a positive IPV screen.^17^ After administering the 5-item primary screening tool (HITS), clinicians must manually record whether the primary screen result was positive or negative. If clinicians select positive, a secondary screen for IPV-related lethality risk is automatically triggered. If they select negative, the secondary screen does not appear, regardless of any positive responses to the primary screening items. Secondary screening for IPV-related lethality includes 3 items from the Danger Assessment^29^: “Has the IPV behavior increased in frequency/severity in the past 6 months?” “Has your partner ever choked or strangled you?” and “Do you believe your partner may kill you?” Positive endorsement of any secondary screening item indicates a positive screen for IPV-related lethality risk.

We extracted from the EHR sex (male or female), age, race (American Indian or Alaska Native, Asian, Black or African American, Native Hawaiian or Pacific Islander, White, multiracial, or unknown), ethnicity (Hispanic/Latino, non-Hispanic/Latino, or unknown), marital status (married, unmarried, or unknown), service-connected disability status (ie, a rating that reflects the severity of one’s service-connected disability and its impact on their ability to work and perform daily activities^33^ [no service connection, ≤50%, >50%, or other (aid and attendance, Purple Heart recipient, or VA pension)]), and housing status (homeless or unstably housed or stably housed). We included race and ethnicity, along with other sociodemographic characteristics, to assess potential disparities across patient populations and associations with screening outcomes. These variables are recorded in the EHR based on patient self-report or prior patient records.

### Statistical Analysis

Analyses were conducted from January 8 to June 18, 2025. We used descriptive statistics to assess rates of protocol-concordant lethality screening administration, IPV-related lethality risk, and item-level endorsement of lethality screening items. To assess rates of lethality screening administration, we identified cases in which patients were documented as having a negative screen in the EHR but should have been classified as positive for IPV per VHA guidelines (due to having reported at least 1 item on the modified HITS primary IPV screen). We first examined associations between sex and administration of the lethality screening tool for all patients who endorsed at least 1 item on the modified HITS and then associations between sex and a positive screen for IPV-related lethality among those who were screened for IPV-related lethality risk. Finally, we examined associations between sex and endorsement of item-level responses among the patients who were screened for IPV-related lethality. Multilevel mixed-effects logistic regression, with random intercepts to account for clustering within facilities, controlling for race, ethnicity, marital status, service-connected disability status, and housing status, was used to examine associations between sex and administration of lethality screening, positive screens for IPV-related lethality risk, and item-level responses on the lethality screening tool.

All analyses were conducted using Stata/MP, version 18 (StataCorp LLC). All models were within range (variance inflation factor mean [range], 1.23 [1.01-1.87]; tolerance range, 0.53-0.99). We set the statistical significance level at *P* < .05 and used 2-tailed tests. All observations had full data.

## Results

### Descriptive Characteristics

**Figure 1.**
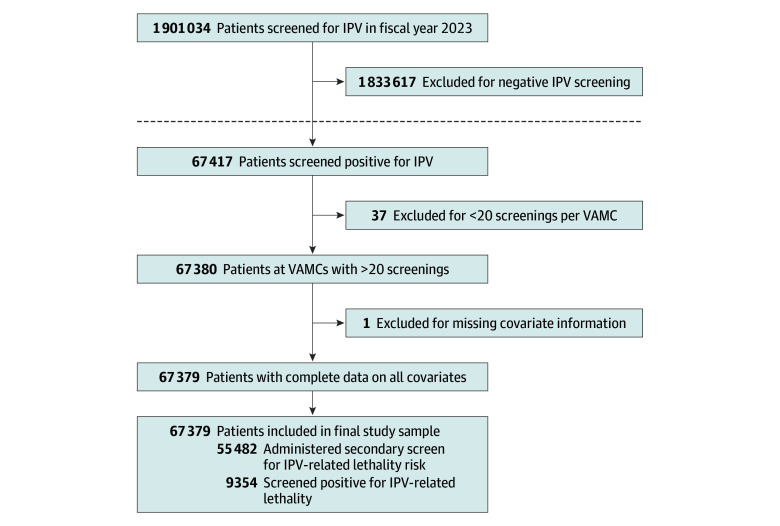
Study Cohort Selection Criteria The full PRISM (Partnered Evaluation of Relationship Health Innovations and Services Through Mixed Methods) cohort is represented above the dashed line, and patient data used for this study are shown below the dashed line. IPV indicates intimate partner violence; VAMC, Veterans Affairs medical center.

After removing veterans who did not endorse at least 1 item on the modified HITS (n = 1 265 115), did not meet criteria for screening adoption (defined as screening at least 20 patients at the facility^16^ [n = 37]), and had missing data on sex (n = 1), the final analytic sample included 67 379 patients from 131 VA medical centers (Figure 1). The mean (SD) age of the sample was 52.3 [16.1] years, and 23.0% were women and 77.0% men. By race, 0.9% of patients were identified as American Indian or Alaska Native, 1.1% as Asian, 23.0% as Black or African American, 0.9% as Native Hawaiian or Pacific Islander, 62.9% as White, 1.4% as multiracial, and 9.8% as unknown. By ethnicity, 8.2% were identified as Hispanic/Latino, 82.4% as non-Hispanic/Latino, and 7.1% as unknown. More participants were married (58.3%) than unmarried (39.5%), and marital status was unknown for 2.2% of patients. Most patients (69.0%) had a service-connected disability rating greater than 50%, 15.0% were rated as 50% or less, 15.3% were rated as having no disability, and 0.7% reported another form of service-connected disability. Additionally, 89.5% of patients were stably housed, and 10.5% were homeless or unstably housed (Table 1).

**Table 1.** Patient Characteristics, IPV Screening Rates, and Associations With Administration of the IPV-Related Lethality Screening Tool

Characteristic	Screened positive for IPV, No. (%)	Administered IPV-related lethality screen, No. (%)	AOR (95% CI)	*P* value^a^
No.	67 379 (100)	55 482 (82.3)	NA	NA
Age, mean (SD), y	52.3 (16.1)	51.7 (15.9)	0.99 (0.99-0.99)	<.001
Sex				
Male	51 862 (77.0)	41 837 (80.7)	1 [Reference]	<.001
Female	15 517 (23.0)	13 645 (87.9)	1.42 (1.33-1.51)	
Race				
American Indian or Alaska Native	602 (0.9)	494 (82.1)	1.16 (0.93-1.45)	<.001
Asian	716 (1.1)	571 (79.7)	1.17 (0.87-1.55)	
Black or African American	15 504 (23.0)	13 219 (85.3)	0.81 (0.61-1.08)	
Native Hawaiian or Pacific Islander	633 (0.9)	550 (86.9)	0.99 (0.79-1.25)	
White	42 358 (62.9)	34 397 (81.2)	1 [Reference]	
Multiracial	938 (1.4)	806 (85.9)	1.35 (0.98-1.86)	
Unknown	6628 (9.8)	5445 (82.2)	0.97 (0.78-1.21)	
Ethnicity				
Non-Hispanic/Latino	57 070 (84.7)	47 015 (82.4)	1 [Reference]	.79
Hispanic/Latino	5546 (8.2)	4549 (82.0)	1.06 (0.97-1.15)	
Unknown	4763 (7.1)	3918 (82.3)	0.95 (0.86-1.05)	
Marital status				
Married	39 286 (58.3)	31 912 (81.2)	1 [Reference]	<.001
Unmarried	26 614 (39.5)	22 352 (84.0)	1.03 (0.98-1.07)	
Unknown	1479 (2.2)	1218 (82.4)	0.98 (0.85-1.14)	
Service-connected disability status				
No disability	10 321 (15.3)	8127 (78.7)	1 [Reference]	<.001
≤50	10 109 (15.0)	8032 (79.5)	1.03 (0.96-1.10)	
>50	46 494 (69.0)	38 964 (83.8)	1.25 (1.18-1.32)	
Other^b^	455 (0.7)	359 (78.9)	0.95 (0.75-1.21)	
Housing status				
Stably housed	60 279 (89.5)	49 185 (81.6)	1 [Reference]	<.001
Homeless or unstably housed	7100 (10.5)	6297 (88.7)	1.58 (1.46-1.71)	

Abbreviations: AOR, adjusted odds ratio; IPV, intimate partner violence; NA, not applicable; VA, US Department of Veterans Affairs.

^a^ Difference tests used Pearson χ^2^ goodness-of-fit test for all variables except for age, which used logistic regression.

^b^ Includes aid and attendance, Purple Heart recipient, or VA pension.

### Administration of IPV-Related Lethality Risk Screening

All 131 VA medical facilities implemented the secondary screen for IPV-related lethality risk. Of the 67 379 patients who endorsed at least 1 item on the primary IPV screen, 55 482 (82.3%) were documented as IPV positive, and 11 897 reported IPV but were misclassified as negative and not administered the IPV-related lethality risk screen, yielding a false-negative rate of 17.7% (Figure 1; Table 2). Women were 106% more likely to be administered the IPV-related lethality risk screen compared with men (relative risk ratio [RRR], 1.06; adjusted odds ratio [AOR], 1.42 [95% CI, 1.33-1.51]). Specifically, 10 025 men (19.3%) were recorded as IPV negative and thus, not administered the secondary screen compared with 1872 women (12.1%). An additional 429 patients (0.1%) were recorded as IPV positive but were also not administered the secondary screen.

**Table 2.** Administration and Endorsement of IPV-Related Lethality Screening

Assessment item	Patients, No. (%)			*P* value^b^
	Total (N = 67 379^a^)	Male (n = 51 862)	Female (n = 15 517)	
Protocol-concordant administration of IPV lethality screen				
No	11 897 (17.7)	10 025 (19.3)	1872 (12.1)	<.001
Yes	55 482 (82.3)	41 839 (80.7)	13 644 (87.9)	
Overall lethality risk				
Negative	46 128 (83.1)	36 288 (86.7)	9840 (72.1)	<.001
Positive	9354 (16.9)	5551 (13.3)	3804 (27.9)	
Increase in IPV frequency or severity				
Not endorsed	47 847 (86.2)	36 941 (88.3)	10 906 (79.9)	<.001
Endorsed	7635 (13.8)	4896 (11.7)	2739 (20.1)	
Strangulation by partner				
Not endorsed	53 243 (96.0)	41 135 (98.3)	12 108 (88.7)	<.001
Endorsed	2239 (4.0)	702 (1.7)	1537 (11.3)	
Fear of homicide				
Not endorsed	53 429 (96.3)	40 906 (97.8)	12 523 (91.8)	<.001
Endorsed	2053 (3.7)	931 (2.2)	1122 (8.2)	

Abbreviation: IPV, intimate partner violence.

^a^ Patients with at least 1 endorsement of IPV on the primary screen were eligible for the secondary screen for IPV-related lethality.

^b^ Differences calculated using Pearson χ^2^ goodness-of-fit test for all variables except for age, which was calculated using logistic regression.

### IPV-Related Lethality Screening Outcomes

**Figure 2.**
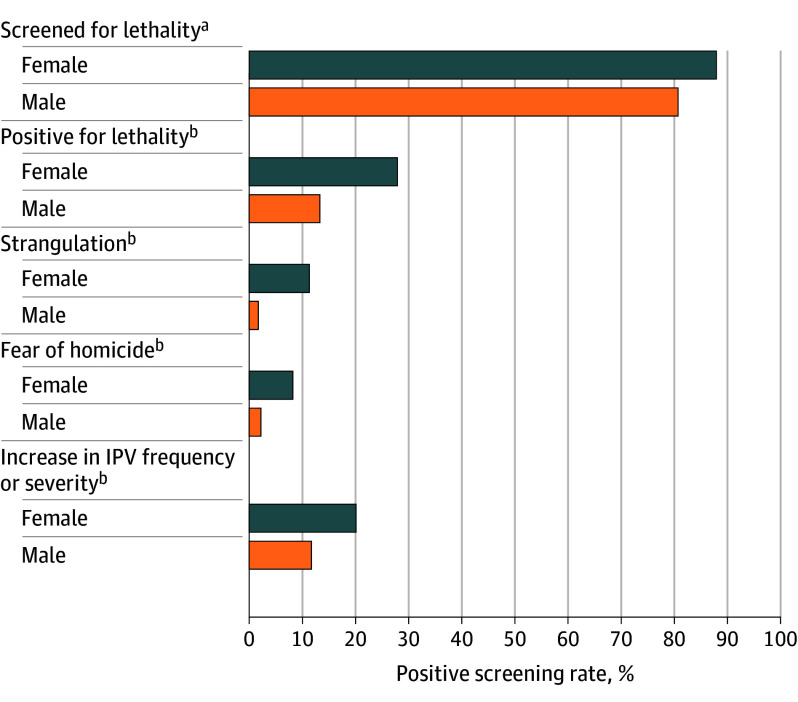
Protocol-Concordant Intimate Partner Violence (IPV) Lethality Screening Administration, Screening Outcomes, and Endorsement of Lethality Screening Items ^a^Rates calculated from the total number of men (51 862) and women (15 517) who screened positive for IPV. ^b^Rates calculated from the total number of men (41 837) and women (13 645) administered the IPV-related lethality screen.

Among the 55 482 patients screened for IPV-related lethality risk, 9354 (16.9%) endorsed past-year lethality risk based on an affirmative response to at least 1 of the 3 items. Women were 195% more likely to screen positive for IPV-related lethality risk compared with men (RRR, 1.95; AOR, 2.29 [95% CI, 2.16-2.41]) (Table 3), with 3804 (27.9%) women and 5551 (13.3%) men reporting past-year risk of IPV-related lethality (Figure 2). Compared with men, women were 515% more likely to report their partner choking or strangling them in the past 6 months (RRR, 5.15; AOR, 5.55 [95% CI, 5.02-6.14]), believing that their partner may kill them (315% more likely; RRR, 3.15; AOR, 3.31 [95% CI, 2.99-3.66]), and an increased frequency or severity of IPV behaviors in the past 6 months (161% more likely; RRR, 1.61; AOR, 1.76 [95% CI, 1.65-1.86]) (Table 3). A total of 702 (1.7%) men reported strangulation and 931 (2.2%) reported fear of being killed by their partner (Table 2).

**Table 3.** Item-Level Rates and Associations With IPV-Related Lethality Screening

Variable	Overall lethality risk (n = 55 482)		Strangulation by partner (n = 2239 [4.0%])			Fear of homicide (n = 2053 [3.7%])			Increased frequency or severity (n = 7635 [13.8%])		
	AOR (95% CI)	*P* value	Positive, No. (%)	AOR (95% CI)	*P* value	Positive, No. (%)	AOR (95% CI)	*P* value	Positive, No. (%)	AOR (95% CI)	*P* value
Age	1.00 (1.00-1.00)	<.001	42.8 (13.3)	0.98 (0.98-0.98)	<.001	47.5 (14.4)	1.00 (1.00-1.01)	.11	49.5 (15.4)	1.00 (1.00-1.00)	.34
Sex											
Male	1 [Reference]	NA	702 (31.4)	1 [Reference]	NA	931 (45.3)	1 [Reference]	NA	4896 (64.1)	1 [Reference]	NA
Female	2.29 (2.16-2.41)	<.001	1537 (68.6)	5.55 (5.02-6.15)	<.001	1122 (54.7)	3.31 (2.99-3.66)	<.001	2739 (35.9)	1.76 (1.65-1.86)	<.001
Race											
American Indian or Alaska Native	1.06 (0.83-1.34)	.66	29 (1.3)	0.87 (0.58-1.31)	.50	27 (1.3)	0.83 (0.55-1.26)	.39	68 (0.9)	1.13 (0.86-1.47)	.39
Asian	1.13 (0.84-1.52)	.42	36 (1.6)	0.77 (0.47-1.28)	.32	24 (1.2)	0.67 (0.39-1.14)	.14	77 (1.0)	1.24 (0.90-1.72)	.19
Black or African American	0.92 (0.66-1.28)	.63	735 (32.8)	0.91 (0.53-1.56)	.73	697 (34.0)	0.77 (0.43-1.39)	.39	2047 (26.8)	0.97 (0.68-1.39)	.88
Native Hawaiian or Pacific Islander	0.88 (0.69-1.14)	.34	16 (0.7)	0.70 (0.45-1.08)	.11	18 (0.9)	0.71 (0.46-1.10)	.13	63 (0.8)	0.97 (0.73-1.28)	.82
White	1 [Reference]	NA	1144 (51.1)	1 [Reference]	NA	1037 (50.5)	1 [Reference]	NA	4514 (59.1)	1 [Reference]	NA
Multiracial	0.66 (0.47-0.94)	.02	51 (2.3)	0.37 (0.19-0.71)	.003	36 (1.8)	0.50 (0.27-0.95)	.03	138 (1.8)	0.78 (0.53-1.13)	.18
Unknown	0.96 (0.76-1.22)	.74	228 (10.2)	0.69 (0.46-1.03)	.07	214 (10.4)	0.65 (0.43-0.97)	.03	728 (9.5)	1.08 (0.83-1.41)	.57
Ethnicity											
Non-Hispanic/Latino	1 [Reference]	NA	1835 (82.0)	1 [Reference]	NA	1709 (83.2)	1 [Reference]	NA	6433 (84.2)	1 [Reference]	NA
Hispanic/Latino	1.01 (0.92-1.11)	.81	224 (10.0)	1.11 (0.94-1.31)	.21	172 (8.4)	1.08 (0.90-1.30)	.39	627 (8.2)	0.98 (0.88-1.08)	.67
Unknown	1.12 (1.00-1.25)	.05	180 (8.0)	1.01 (0.83-1.24)	.92	172 (8.4)	1.12 (0.91-1.38)	.28	575 (7.5)	1.09 (0.97-1.23)	.16
Marital status											
Married	1 [Reference]	NA	838 (37.4)	1 [Reference]	NA	812 (39.6)	1 [Reference]	NA	3892 (51.0)	1 [Reference]	NA
Unmarried	1.19 (1.13-1.25)	<.001	1356 (60.6)	1.38 (1.25-1.52)	<.001	1201 (58.5)	1.47 (1.33-1.62)	<.001	3575 (46.8)	1.09 (1.03-1.15)	
Unknown	1.00 (0.85-1.18)	.98	45 (2.0)	1.00 (0.72-1.38)	.99	40 (1.9)	1.04 (0.74-1.46)	.81	168 (2.2)	1.06 (0.89-1.26)	.53
Service-connected disability status											
No disability	1 [Reference]	NA	251 (11.2)	1 [Reference]	NA	244 (11.9)	1 [Reference]	NA	1009 (13.2)	1 [Reference]	NA
≤50	1.00 (0.92-1.10)	.95	217 (9.7)	0.83 (0.69-1.01)	.06	216 (10.5)	0.95 (0.79-1.15)	.59	954 (12.5)	0.98 (0.89-1.08)	.75
>50	1.17 (1.09-1.26)	<.001	1763 (78.7)	1.04 (0.90-1.20)	.59	1573 (76.6)	1.26 (1.09-1.46)	.002	5605 (73.4)	1.15 (1.07-1.24)	<.001
Other^a^	1.26 (0.95-1.66)	.10	8 (0.4)	0.70 (0.34-1.45)	.34	20 (1.0)	1.41 (0.87-2.28)	.17	67 (0.9)	1.37 (1.04-1.82)	.03
Housing status											
Stably housed	1 [Reference]	NA	1542 (68.9)	1 [Reference]	NA	1408 (68.6)	1 [Reference]	NA	6020 (78.9)	1 [Reference]	NA
Homeless or unstably housed	2.43 (2.28-2.59)	<.001	697 (31.1)	2.97 (2.68-3.29)	<.001	645 (31.4)	3.14 (2.83-3.49)	<.001	1615 (21.1)	2.19 (2.05-2.34)	<.001

Abbreviations: AOR, adjusted odds ratio; IPV, intimate partner violence; NA, not applicable; VA, US Department of Veterans Affairs.

^a^ Includes aid and attendance, Purple Heart recipient, or VA pension.

## Discussion

This cross-sectional study of sex differences in IPV-related lethality screening administration, outcomes, and response patterns found that fidelity to IPV-related lethality screening administration was lower among men than women. In addition, among patients screened for lethality, women were more likely than men to have positive lethality screens. The VHA has made substantial investments and progress in implementing health care screenings as a crucial step for connecting patients to care,^34,35^ including screening and response to IPV.^17^ However, the implementation of IPV screening and response has shown considerable variation across the health care system,^16,18,19,20,21^ and previous efforts have focused on examining IPV screening implementation among female patients.

Our study builds on the only other study to date that evaluated the implementation and effectiveness of IPV-related lethality screening among veterans.^15^ This prior work, conducted exclusively in women in the early years of VHA’s IPV screening rollout, found low adoption of the lethality screen, with only 3 of 11 VA medical centers (27.3%) implementing the lethality screening tool and only 56.4% of eligible women being screened.^15^ Our study found a substantial increase in IPV-related lethality screening adoption as the rollout has continued and expanded to men, as reflected by 100% of facilities screening for IPV-related lethality in fiscal year 2023 and an 82.3% implementation rate overall following a positive IPV primary screen (87.9% among women and 80.7% among men). This increase in adoption is encouraging, as uptake in IPV-related lethality screening and response can link high-risk patients to critical and potentially lifesaving follow-up care.^36^ Future studies that examine screening practices associated with system-level factors, such as practitioner type, facility differences, leadership support, and training, may be valuable in offering additional context for understanding screening variation across sites and optimizing screening implementation.

Although adoption of IPV-related lethality screening has improved across the health care system, fidelity of implementation varied by sex. Our study responds to the USPSTF’s call for research on the accuracy of screening men for IPV and examining effectiveness of screening among men.^8^ Our findings revealed that men who endorsed 1 or more items on the primary screen were more likely than women to be inaccurately classified as negative for IPV (19.3% vs 12.1%), highlighting a notable sex disparity. Consequently, men were erroneously identified as IPV negative despite reporting IPV experiences, resulting in clinician failure to administer the IPV-related lethality screen per protocol. Specifically, the 10 025 men who reported past-year IPV were misclassified and not administered the secondary screen for IPV-related lethality (compared with 1872 women). The issue of men being misclassified as negative for IPV on the primary screen may be attributed to clinician bias, though additional research is needed to assess causes for this disparity. Interviews with professionals providing domestic abuse services have revealed a lack of acknowledgment of men’s IPV experiences and a prevailing view that IPV is primarily a heteronormative, women-specific issue.^28^ Such beliefs may prevent clinicians from recognizing and accepting the occurrence of IPV among men and may deter men from sharing their IPV experiences. In another study among men with IPV experiences, most reported not seeking help for IPV due to shame, distrust of formal support services, and past experiences with formal IPV support services as unhelpful.^23^ Other reasons clinicians may not conduct IPV-related lethality screening may include previously identified barriers to IPV screening in the health care system, such as time constraints, discomfort in addressing IPV, and system-level challenges (eg, limited resources, lack of training, competing patient care priorities).^16,18,19^

The primary IPV screening instrument is not a diagnostic tool; it is designed to prompt further assessment. As such, it is possible that patient-clinician discussion following positive endorsement of an item on the primary IPV screen may lead to clinical determinations that a patient is IPV negative and the clinician bypassed the secondary screen. For instance, if a patient reported that their partner yelled at them but then clarified that raising their voice was necessary due to hearing difficulties, or a patient expressed that they did not view their partner’s behavior as IPV, the clinician may have classified the screen as negative. Differences in how IPV is conceptualized may contribute to this issue as well, as men may be less likely to recognize nonphysical IPV behaviors as abuse.^37^ However, further research is needed to evaluate whether sex differences exist in the overendorsement or underendorsement of IPV to others, including clinicians. Moreover, the HITS tool was developed and validated using samples of women who experienced IPV, and research is needed to test whether it performs as effectively in men.^11,12,13^ Further efforts are also needed to understand clinician decision-making and action, as well as potential bias or prejudice, with adherence to IPV protocols, particularly at the screening stage in which the goal is to ensure adequate detection and follow-up care, emphasizing sensitivity over specificity to avoid missing patients who potentially need lifesaving services. As such, additional training for clinicians aimed at acknowledging women and men’s experiences with IPV and responding appropriately without minimizing the abuse, as possibly observed in this study, might be beneficial.

Although it is common for both partners in a relationship to experience and use IPV,^38^ particularly among military and veteran couples,^39,40,41^ and veteran men report experiencing past-year IPV at rates comparable to women,^22^ the outcomes of IPV or motivation to use violence^42^ are not necessarily equivalent across sexes. Women experience more severe forms of violence and more health-related outcomes, including fear and safety concerns and psychological and physical injuries, than men.^43,44,45^ We found similar trends in that women were nearly twice as likely to experience past-year IPV-related lethality risk compared with men overall and 5.15 times more likely to experience strangulation, 3.15 times more likely to believe their partner might kill them, and 1.61 times more likely to report recent increases in violence frequency or severity. These increased rates of severe and potentially lethal IPV among women compared with men also align with findings highlighting sex differences in intimate partner homicide risk and support the value of identifying and responding to IPV in the health care system.^14^

Although women experience higher rates of injury and potentially lethal IPV than men, men face these dangers as well. In one help-seeking sample of men who experienced female-perpetrated physical IPV, more than 70% reported injuries, of which more than 40% required medical intervention.^46^ Our study did not specifically measure injury; nevertheless, we found that 702 (1.7%) men reported strangulation and 931 (2.2%) reported fear of being killed by their partner. Research on IPV-related strangulation among men is notably lacking, though nonfatal strangulation is a robust risk factor for intimate partner homicide with consequent long-term negative mental and physical health outcomes.^47^ To our knowledge, this study is the first to investigate the implementation and use of these IPV-related lethality risk items among men, advancing research in this high-risk area and emphasizing the need for further studies, particularly with male patients.^14,29^

### Limitations

This study had some limitations. First, as this was a retrospective analysis of EHR data, we did not have additional information to contextualize findings, such as why patients who endorsed IPV on the primary screening were misclassified. Second, EHR documentation may include errors or not represent prevalence due to barriers to disclosure or documentation concerns. Data were drawn from clinical encounters within VHA, a health care system with national policies, programs, and implementation strategies supporting IPV screening and response. As such, the findings may not be transferable to other patient populations and health care systems as lack of supports are known barriers to IPV screening and response implementation.^48,49^ Nonetheless, VHA is the largest integrated health care system in the US, and program evaluation efforts within this health care system may serve as a model for informing quality improvement within other health care settings. Finally, our findings do not represent prevalence of IPV or IPV-related lethality as we know that both women and men experience barriers to IPV disclosure within the health care setting; as such, we were limited by potential underreporting of IPV experiences.

## Conclusions

This cross-sectional study of sex differences in IPV-related lethality risk screening found differences between men and women in the administration, outcomes, and response patterns of IPV-related lethality screening among VHA patients. Nearly 1 in 5 men and 1 in 8 women were misclassified as IPV negative despite reporting IPV during screening and, thus, were not administered the IPV-related lethality screening assessment. Of those screened, women had higher lethality risk compared with men, with nearly one-third reporting strangulation, fear of homicide, or recent increases in IPV frequency and/or severity. Men also reported lethality risk, and their IPV experiences and associated health outcomes should not be ignored. Continued research is needed to identify effective strategies for connecting these patients at risk for IPV lethality to services that support their safety and healing. These findings may inform clinical and implementation strategies to ensure that patients who are screened for IPV receive appropriate follow-up screening and response care essential to improving patient health and safety outcomes.

## Notes

Supplement.
Data Sharing Statement
