# Supplementary material for: Sex Differences in Intimate Partner Violence Lethality Risk Screening Administration and Outcomes
Source: JAMA Netw Open. 2025 Dec 3;8(12):e2545519. doi: 10.1001/jamanetworkopen.2025.45519 (PMC12676351; doi:10.1001/jamanetworkopen.2025.45519)
Supplement: Supplement. — Data Sharing Statement [file jamanetwopen-e2545519-s001.pdf]

Data Sharing Statement

Portnoy. Sex Differences in Intimate Partner Violence Lethality Risk Screening Administration and Outcomes. *JAMA Netw Open*. Published November 25, 2025.
doi:10.1001/jamanetworkopen.2025.45519

Data
Data available: No

Additional Information
Explanation for why data not available: Those interested in the deidentified dataset and data dictionary underlying this manuscript can send an email request to the corresponding author: galina.portnoy@va.gov. Data sharing will be required to comply with government procedures and can only be shared a signed data access agreement.
